# Xuemei Chen: Original innovation often derives from free exploration

**DOI:** 10.1093/nsr/nwae179

**Published:** 2024-05-23

**Authors:** Chao Gu

**Affiliations:** Research Center for Chinese Politics, Peking University, China

## Abstract

Xuemei Chen is a distinguished biologist. She was elected a member of the US National Academy of Sciences in 2013, and is now the Dean of the School of Life Sciences at Peking University. Her main research areas are flower development, plant microRNAs, and RNA modifications. In this interview, Xuemei Chen discussed the prospects of plant research, and emphasized the significance of free exploration to achieve the ground-breaking ‘0 to 1’ innovation—until free exploration leads to the discovery of the ‘1’, one may not know what the ‘0’ is and may not be able to intentionally seek it. She also introduced the upcoming Beijing Advanced Center of RNA Biology (BEACON), and expressed her views on organized scientific research.

## Teaser

An interview with Xuemei Chen, a distinguished plant biologist, Dean of the School of Life Sciences at Peking University, and a member of the US National Academy of Sciences.

## PROSPECTS FOR PLANT RESEARCH

***NSR:*** Compared to animal small RNAs, what are the unique behaviors and functions of plant small RNAs?

***Chen:*** Evolution is quite interesting. Scientists largely agree that RNA interference (RNAi), a gene silencing mechanism, existed before the evolutionary divergence of animals and plants, and RNAi employs a type of small RNA known as small interfering RNAs (siRNAs). microRNAs (miRNAs), a type of small RNA similar to siRNAs in biogenesis and modes of action, show no homology in sequence in plants and animals, which might be a result of the rapid evolution of miRNA genes. However, their mechanisms of production and function are similar in plants and animals. Within the family of plants, take the monocot rice and the dicot *Arabidopsis thaliana* as examples, they both have hundreds of miRNAs, but the number of their shared miRNAs is only around 20. It means that miRNA sequences evolved rapidly among plant lineages.

***NSR:*** Do plant and animal small RNAs require different research methods?

***Chen:*** The methods are similar—we use a plant model organism to do research on small RNAs. We found that plant small RNAs are methylated, so we set out to see whether animal small RNAs are similarly modified. The small number of animal miRNAs we tested were not methylated and it turned out that most animal microRNAs are not methylated. But animal piRNAs and some siRNAs were found by others to be similarly modified. Therefore, plant studies can sometimes guide research in animals, as biology is an integrative research discipline and many basic molecular mechanisms are conserved.

***NSR:*** Currently, what are the most important research directions in plant science?

***Chen:*** First, plants have undergone large-scale genetic changes during evolution, such as polyploidization. Humans and most animals are diploid, but some plants can be tetraploids or hexaploids. This area is still under-researched. From the genomic evolution perspective, this is an issue quite unique to the field of plant science.

**Figure 1:**
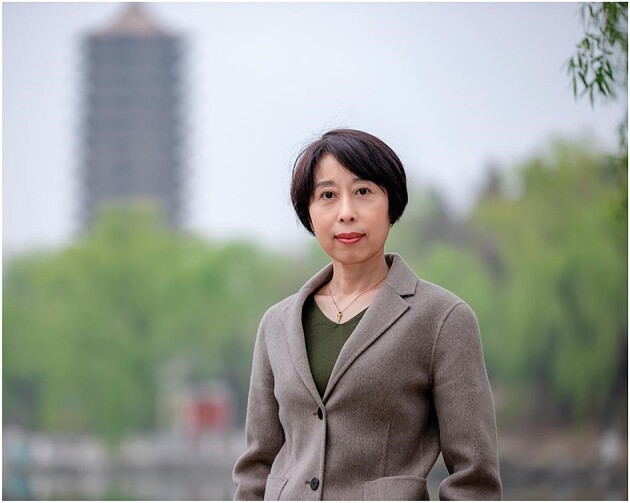
Prof. Xuemei Chen in Peking University. *(Courtesy of Prof. Chen)*

Second, some phenomena in plants are still poorly studied, such as the symbiotic relationships between plants and microorganisms. For example, how microorganisms such as mycorrhizae interact with plant roots or even mediate interactions between roots of different plants is an area that is worth investigating.

Third, secondary metabolism related to synthetic biology is an important topic. Plants have primary metabolism like animals, but also have many secondary metabolites, mainly to attract or repel insects. Some of these secondary metabolites are the effective molecules of traditional Chinese medicine. This is a broad research area with many under-explored topics.

Fourth, plastids are fascinating and important organelles, with chloroplasts being only one type of plastids. The development of different types of plant plastids such as amyloplasts, chromoplasts, etc. from a ‘stem-cell’-like state is poorly understood. There are still many outstanding questions in plastid biology, even with chloroplasts that have been long studied.

Fifth, plant nutrition, particularly the absorption, transport, and utilization of nitrogen and phosphorus, is relevant to agriculture and a lasting topic of investigation.

Last, many plant species remain un- or underexplored. I have been mainly studying the model plant *Arabidopsis* in the past. After returning to China, as a ‘new’ start, I looked for another model plant to study besides *Arabidopsis*, and finally decided to work on soybean because it is important for agriculture but many biological phenomena with this plant species are not yet understood at the molecular level. I am particularly interested in the reproductive development of soybean and hope to enable hybrid breeding in the future.

***NSR:*** What are the current research interests of your lab?

***Chen:*** It has been over 20 years since the discovery that small RNAs are prevalent in plants. The basic mechanisms of plant small RNAs have been largely deciphered, and this field has reached a plateau in terms of fundamental discoveries. About 5 to 6 years ago, I began looking for a new research direction, and we started to work on RNA modifications. We are currently focusing on a broad category of modifications at the 5′ termini of RNA molecules, referred to as RNA caps.

## ‘0 TO 1’ INNOVATION AND FREE EXPLORATION

***NSR:*** What prompted you to enter the field of plant miRNAs over 20 years ago?

***Chen:*** Initially, I studied flower development, devoted to understanding which genes control the differentiation and growth of floral organs. I used forward genetics methods—creating mutants through large-scale random mutagenesis and screening for mutants with floral defects followed by the identification of the corresponding genes. I discovered several genes crucial for flower development. Then I realized that one of these genes, *HEN1*, might be related to miRNAs. Why did I think so? Because a colleague in the lab at Caltech, where I did my postdoctoral research, worked on a plant gene *DICER-LIKE1* (*DCL1*) that was later found to be a homolog of the nematode gene *dicer*, and the phenotype of *hen1* mutants resembled that of *dcl1* mutants. Dicer was shown to produce two small RNAs (lin-4 and let-7) in *C. elegans*, so I hypothesized that *HEN1* could probably produce small RNAs as well. At that time, miRNAs were not known to exist in plants, and I decided to take a risk and see if they existed. The risk wasn't too great because in 1999, it was found that the gene silencing (RNAi) process in plants could produce similar small RNAs known as siRNAs. siRNAs were derived from exogenous sources such as transgenes, and our study tried to find endogenous miRNAs. We indeed found them and also experimentally proved that without *HEN1*, the production of miRNAs would significantly decrease, confirming that *HEN1* is indeed a gene that produces miRNAs. We continued to study the connection between *HEN1* and flower development and found that a particular miRNA was crucial for floral development. Therefore, the discovery of miRNAs from plants, at least in my lab, was not intentional.

Actually, the discovery of miRNAs in animals was a similar story. The earliest known miRNA was discovered in 1993 by Victor Ambros in nematodes. He, like us, was studying development

Only after discovering ‘1’ did we know where ‘0’ was.

—Xuemei Chen

through random mutations and discovered a gene mutation that inhibited nematode development from larval stage 1 to stage 2. After identifying this gene, it was found to encode a 21–22 nt small RNA. It took another eight years before it was realized that miRNAs are ubiquitous in animals.

Now, Chinese scientists and government officials often talk about ‘from 0 to 1’ in terms of original innovation. But considering the discovery of miRNAs, it was not a case that we knew miRNAs were hidden from us and then searched for them. Instead, we didn't even know miRNAs existed. That is, only after discovering ‘1’ did we know where ‘0’ was. The previously unknown entity was ‘0’. ‘From 0 to 1’ is definitely not about knowing ‘0’ and seeking for ‘1’. Many fundamental scientific discoveries are not intentionally made but only occur through long-term accumulation and observation in the research process by connecting one dot to another and then moving to the next. That is how breakthroughs emerge.

***NSR:*** Is your research on the methylation mechanism of miRNAs an original ‘0 to 1’ innovation?

***Chen:*** I don't think it's a real ‘from 0 to 1’, but the discovery is still quite important. We found that unmodified small RNAs are unstable in vivo. In terms of small RNA drugs, this discovery is significant because it tells us small RNA-based drugs should be modified.

***NSR:*** Have you been deeply involved in this field purely out of interest? Does this area of research have potential applications?

***Chen:*** Yes, I'm driven by interest. I originally studied flower development, which is a very basic biological research direction with little practical use beyond horticulture. Then my research on flower development led to the discovery of the existence of miRNAs in plants. Once miRNAs were discovered there were many unknown mechanisms to be studied, such as how miRNAs are produced, how they function, and which aspects of plant biology they regulate. So I decided to shift my research focus from flower development to miRNAs and have been working in this field for 20 years. Plant miRNA research can certainly lead to potential applications. For example, miRNAs affect plant traits, such as flowering time, fruit size and disease resistance. Additionally, male sterility lines are important in plant breeding, and several miRNAs play key roles in the development of plant male reproductive organs and have significant application value. However, my past research has not focused on technology and applications, but on basic science.

***NSR:*** You’ve recently shifted from miRNAs to RNA modifications. What prompted this change?

***Chen:*** It's also driven by interest. This interest originally stemmed from my doctoral research. During my PhD, my advisor was very interested in molecular processes at the RNA level. In the early 1990s, most people were studying transcription, but my advisor focused on post-transcriptional processes. I became interested in these processes at that time. Later, when I was a postdoc, my lab focused on flower development research, and most genes known to regulate flower development at the time were transcription factors. I wondered why post-transcriptional processes weren't important in flower development. Actually, it wasn't that they were unimportant, but that there were gene redundancies in those processes that precluded their discovery by forward genetics. When I continued research on flower development in my own group, we found new genes that regulate flower development and they were almost all related to RNAs, and as a result, I actually returned to the direction of my doctoral studies. So, I’ve always been particularly interested in the biology of RNAs.

In recent years, the field of miRNAs became a mature field and I wanted to find a new research direction in RNA biology. I didn't want to study RNA splicing, RNA degradation, translation or other processes that had been studied for many years, although there still remain outstanding questions in these processes, but I was attracted to RNA noncanonical capping upon reading the literature. The capping of RNA by certain cellular metabolites, such as NAD, dpCoA, FAD, etc., has been found to be ubiquitous in prokaryotes and eukaryotes (animals, fungi and plants), and there are enzymes that remove them, suggesting they are subject to some level of regulation. The next step is to study what the modifications do. Of course, such research relies upon technological advances and I think the current technology is capable of exploring these RNA modifications.

***NSR:*** What are the main technologies involved?

***Chen:*** The most important one is nucleic acid mass spectrometry. We need to break RNAs into nucleotides to detect RNA modifications. The core facility at Peking University is quite rudimentary in terms of nucleic acid mass spectrometry, so we are in the process of establishing the Beijing Advanced Center of RNA Biology (BEACON), which will include a nucleic acid mass spectrometry facility. Another important technology is omics technology. We need to develop methods that can detect modified RNAs at the transcriptome level. Additionally, AI technology platforms are very important. BEACON is also building an AI platform—not to invest in hardware but to recruit expertise in computational biology.

## RESEARCH MANAGEMENT

***NSR:*** What are the main factors in the success of your miRNA research?

***Chen:*** In terms of my early research, initial funding was very important. The *HEN1* project was supported by March of Dimes, a private foundation dedicated to reduce birth defects, which seemingly had nothing to do with plant research. I proposed a project on flower development, and they funded me because the process in which stem cells differentiate into petals or stamens in the flower is conceptually similar to cell differentiation in early fetuses. They were really open-minded to fund my project, and I am glad that the funded work led to the discovery of a molecular mechanism common to both plants and animals—the chemical modification of small RNAs.

Then I got funding from the federal government. Before receiving the first grant from the US National Science Foundation (NSF) or the US National Institute of Health (NIH), it was permissible to simultaneously submit applications to both institutions. I did so and the application was deemed fundable by both agencies. As NIH provided more funding than NSF, I chose NIH's funding, which was about $200 000 in direct costs, enough to hire one student and two postdocs at the time. These fundings together with the startup fund were crucial to my research during my early stage of being an independent principal investigator (PI).

At that stage, I also conducted experiments myself. From what I've observed, assistant professors in the US, compared to those in China, spend more time doing experiments themselves. Assistant professors in China spend more time away from the bench. For example, a heavy teaching load is not conducive to biological research, because biological experiments require blocks of focused time. When I was an assistant professor, I was in a research institute where I only needed to teach half a course a year, so I could spend most of my efforts on scientific research, just like when I was a postdoc. I was able to stay all day in the lab and communicate with my students in a timely manner.

***NSR:*** After returning to China, what are your feelings about the scientific culture and research management?

***Chen:*** The academic atmosphere at Peking University is very good, with many opportunities for scientific exchanges between labs and with outside groups, both national and international. However, I also find some circumstances in China quite disturbing. There are some large-scale academic conferences in China that invite many distinguished scientists to attend. But they usually only appear in the morning of the first day, and substantive academic exchanges are rare. I have to say that this aspect of the scientific culture is not appealing.

In terms of management, organized scientific research is now emphasized in China. To solve a large engineering problem or build a large device, organized scientific research is certainly a feasible route. But for cutting-edge explorations in biology, it's unclear to me how effective organized scientific research is. Of course, interdisciplinary collaboration and the use of AI and other technologies are very useful and the integration of interdisciplinary expertise may, to some extent, be considered organized research, but biological research still depends on scientists’ interest-based free explorations to produce the so-called ‘0 to 1’ discoveries. The discovery of CRISPR, the basis of the currently widely explored gene editing technologies, as well as the discovery of miRNAs I mentioned earlier, are examples of achieving ‘1’ before knowing ‘0’ and clearly not results of organized research.

***NSR:*** Is biological research in the US organized?

We are exploring modes of ‘organized research’, or a better word would be ‘synergistic research’.

—Xuemei Chen

***Chen:*** Rarely in universities. Research in national laboratories may be organized. I do not know much about how US national laboratories are run, but my understanding is that national laboratories have an overall goal for the PIs and the PIs are still free to choose their specific projects following their own interests. For example, in a national laboratory for bioenergy, PIs are gathered to work on bioenergy-related projects. Although the ultimate goal maybe applied, labs can still do basic research, such as studying the composition of plant cell walls. To transfer science into technology, one needs to know what the biology is before thinking about how to use it, that is why basic research is also needed in organized research.

***NSR:*** What suggestions do you have for advancing basic research in China?

***Chen:*** Currently, the funding for basic research is quite limited in China. For example, the scale of the National Natural Science Foundation of China (NSFC)’s ‘general projects’ funds is relatively small. Biological research is particularly expensive, and the funding level of the ‘general projects’ category is definitely not enough. However, I agree with the design of the NSFC's funding scheme, which allocates more funds to outstanding scientists such as the ‘Distinguished Young Scholars’ program. With limited total funding, basic research indeed needs these scientists working on cutting-edge projects. But we still need to give opportunities to more young talents. They need to be guaranteed enough startup funding during their assistant professor period to focus on research without too many distractions. In my first five years as an assistant professor, I had little financial worries. That was especially important to me.

***NSR:*** The School of Life Sciences at Peking University (PKU) is one of the first institutions in China to implement a tenure-track system. Do you think there is room for improvement in the tenure-track system?

***Chen:*** Our tenure evaluation is completely aligned with, and maybe even more strict than the international norm. For example, in the US, six recommendation letters are usually enough, but at PKU, we require ten. Moreover, we also consider the response rate of review invitations, which is not considered as far as I know in the US. In past years, there have been some researchers at our school who actually did very well in research but were not granted tenure. Some of them stayed at PKU as co-PIs while some transferred to other universities—and some later became NSFC Distinguished Young Scholars or Yangtze River Scholars. This shows that on the one hand, our standard is high, and on the other hand, there may still be room for improvement in the system to keep the outstanding talents.

In terms of improvements, I think it's not necessary to consider the response rate of tenure review invitations. Another point is that now we ask the reviewers to compare the candidate with benchmark scholars, and names of specific scholars are preferred. But generally, reviewers may be unwilling to compare the candidate with specific persons, and I think it is sufficient to ask them whether or not this candidate can obtain tenure at the reviewers’ institution or to rate the candidate's standing in the specific field.

***NSR:*** What's your vision for BEACON?

***Chen:*** On one hand, BEACON focuses on basic research and aims at cutting-edge discoveries, and on the other hand, it hopes to collaborate with industry to generate some applicable technologies. For example, to tackle a certain genetic disease, we need to understand the underlying mechanism, and then propose/develop innovative RNA editing methods to reverse the genetic defects.

In terms of applied research, in agriculture, we particularly focus on two goals. One is increasing plant production, such as alfalfa, a forage plant with high protein content. China currently imports a large amount of soybean, mainly used for feed. If we can increase alfalfa production, it could reduce the pressure of soybean import. The second issue is plant disease control. Severe plant diseases could lead to crop failure, and now with global warming, plant diseases that were originally in the south are also appearing in the north, so plant disease control is particularly important. RNA technologies have the potential to control some plant diseases. In medicine, we would like to provide the conceptual basis for the development of RNA technologies to fight against cancer or age-related diseases.

***NSR:*** In terms of research management, has BEACON adopted any special measures?

***Chen:*** We are exploring modes of ‘organized research’, or a better word would be ‘synergistic research’. The idea is to encourage BEACON PIs to work towards a few common goals. But BEACON PIs are already PIs at Peking University and have their own research interests. We cannot mandate research directions but will try to encourage new efforts towards common goals through funding mechanisms. For example, regarding term assessment, our strategy is different from the Center for Life Sciences (CLS) of Tsinghua and Peking Universities. The employment term of CLS is five years, allowing free exploration within five years, and evaluation is conducted at the end of the term. While BEACON's term is based on projects with evaluations every year. If the progress in a proposed project is not satisfactory, next-year's funding may be reduced. Nevertheless, the pre-tenure and tenure review for assistant professors still follows PKU's system. The main purpose of this assessment method is to encourage PIs to undertake projects that align with the overall goals of BEACON. We are also exploring some new human resource mechanisms, such as recruiting co-PIs for certain projects to empower our applied basic research and technological innovation.

For cutting-edge basic research, we are also considering how to guide PIs to work towards common goals. We can select a cutting-edge direction, which is relatively poorly explored. If we can organize more scientists to work in this direction, breakthroughs can be expected. This is a reasonable idea, but several prerequisites need to be met. First, the direction must be relatively broad, including not just one or two topics. Second, the direction should hold promise for significant future development. Third, as any research direction involves many unknowns, we can only guide the scientists, but cannot demand them to work on certain subjects. We encourage the PIs to explore in this broad direction to form synergies, but they are still free to explore. Therefore, a key question now is: how to determine the cutting-edge direction that we will work together in?

We have already convened three brainstorming meetings with ten BEACON PIs and also held two meetings with scientists outside BEACON who work on AI and compound screening to gather ideas and gradually refine the research directions. We haven't fully determined the directions yet, but we have identified an applied basic research direction, which is to use RNA as a target to screen for small-molecule drugs. Many drugs are small molecules targeting proteins, and after decades of screening performed by the pharmaceutical companies, most protein targets have been explored. Now we would like to try RNA targets, and believe that this direction is promising. BEACON is recruiting talents globally, and we welcome applicants for PI and co-PI positions!

## WOMEN SCIENTISTS

***NSR:*** As a female scientist, have you felt that you need to put in more effort than your male counterparts during your research career?

***Chen:*** It's better now than in the past, but overall, women are still a vulnerable group in society. When I was an assistant professor in the US, a female professor in my institute, Ruth Steward, was 20 years older than me and was particularly nice to me, I think because she really cared about women in science due to her own difficulties as a female scientist. She is an incredible scientist, having published single-authored papers in *Cell* and *Science*. She was a single author because she did not have her own lab for many years. Female scientists of her age really had a hard time.

I also felt this from my own experience as an assistant professor. The institute I was in was in general very supportive of me, but during my third-year mid-term evaluation, I was made aware of my being ‘slow’ in publications despite having three research papers already, which was actually quite good by any standards in biological research. I did not compare notes with male assistant professors in the institute, but I wonder whether there were double standards.

***NSR:*** In the scientific community, there is still a very typical ‘inverted pyramid’ phenomenon, where the higher the

Even from a selfish perspective, men should consider the long-term benefits of having a successful partner.

—Xuemei Chen

educational and career level, the fewer women scientists, especially elite women scientists (such as academicians). What suggestions do you have for this?

***Chen:*** The concept of male superiority and female inferiority still exists in China, and it definitely affects the growth of women scientists. Some female students have told me that they are very interested in doing research, but their families think it's good enough for them to find a job after obtaining their PhD instead of doing a postdoc and aspiring to become a professor. In family division of labor, people still think that taking care of children is a woman's job. When we no longer have any need in discussing the issue of women scientists in the future, it's probably a perfect society by then.

My first suggestion is to strive to change men's perceptions. Actually, men should consider how to share more household responsibilities. For a family, having a mother who is very successful in her career is a valuable influence for the children. So, even from a selfish perspective, men should consider the long-term benefits of having a successful partner, not just focusing on household chores.

My second suggestion is that universities should consider the male-to-female ratio as a mandate during admissions. Top-tier science and engineering schools like MIT have a near 1 : 1 male-to-female ratio in their admitted students, but this is not the case at Peking University, with the ratio being quite alarming for certain disciplines. A university classroom full of male students is unhealthy and also detrimental to the scientific culture of the discipline. You asked me whether policies like affirmative action in the US also bring unfairness—indeed, such policies are felt by some to be disadvantageous to the Asian community as Asian students need to have much higher scores to be admitted to top universities than other ethnic groups, but I still support the policies overall. Society shouldn't be solidified, and opportunities should be given to disadvantaged groups for them to rise.

## FUNDING

This interview is supported by Noncommunicable Chronic Diseases - National Science and Technology Major Program (2023ZD0509601) and National Social Science Foundation Major Project (23&ZD149).

## Notes

Chao Gu is an Assistant Professor at the Research Center for Chinese Politics, Peking University, China

